# Dose and Time-Dependent Selective Neurotoxicity Induced by Mephedrone in Mice

**DOI:** 10.1371/journal.pone.0099002

**Published:** 2014-06-03

**Authors:** José Martínez-Clemente, Raúl López-Arnau, Sonia Abad, David Pubill, Elena Escubedo, Jorge Camarasa

**Affiliations:** Department of Pharmacology and Therapeutic Chemistry (Pharmacology Section) and Institute of Biomedicine (IBUB), Faculty of Pharmacy, University of Barcelona, Barcelona, Spain; Universidade de São Paulo, Brazil

## Abstract

Mephedrone is a drug of abuse marketed as ‘bath salts". There are discrepancies concerning its long-term effects. We have investigated the neurotoxicity of mephedrone in mice following different exposition schedules. Schedule 1: four doses of 50 mg/kg. Schedule 2: four doses of 25 mg/kg. Schedule 3: three daily doses of 25 mg/kg, for two consecutive days. All schedules induced, in some animals, an aggressive behavior and hyperthermia as well as a decrease in weight gain. Mephedrone (schedule 1) induced dopaminergic and serotoninergic neurotoxicity that persisted 7 days after exposition. At a lower dose (schedule 2) only a transient dopaminergic injury was found. In the weekend consumption pattern (schedule 3), mephedrone induced dopamine and serotonin transporter loss that was accompanied by a decrease in tyrosine hydroxylase and tryptophan hydroxylase 2 expression one week after exposition. Also, mephedrone induced a depressive-like behavior, as well as a reduction in striatal D2 density, suggesting higher susceptibility to addictive drugs. In cultured cortical neurons, mephedrone induced a concentration-dependent cytotoxic effect. Using repeated doses for 2 days in an elevated ambient temperature we evidenced a loss of frontal cortex dopaminergic and hippocampal serotoninergic neuronal markers that suggest injuries at nerve endings.

## Introduction

Mephedrone (4-methylmethcathinone) is a synthetic ring-substituted cathinone often marketed as “bath salt”. It appears to be used by people involved in the dance and music scene and also used more broadly by many young adults and adolescents [1]. It is known to have similar effects to other psychostimulant drugs [2], [3] but many users consider the effects of cathinones to be superior to cocaine and MDMA (3,4-methylenedioxymethamphetamine) [4], [5]. Moreover, the abuse potential of cathinone derivatives is comparable with that of cocaine or MDMA [6].

Based in its chemical structure, mephedrone is expected to elicit stimulant effects similar to amphetamines [1], [7]. It has been demonstrated that mephedrone acts as a substrate for monoamine transporters [8], [9], which induces transporter-mediated depolarizing current [10] and releases monoamines by reverse transport [11]. Different authors [12]–[14] have shown that mephedrone administration to rats increases extracellular dopamine (DA) and serotonin (5-HT) in rat brain, similar to the effects of MDMA. All these results evidenced that this drug interacts with DA and 5-HT transporters displaying a similar pattern to other amphetamine derivatives.

After a first dose, mephedrone users evidenced a desire to re-dose, leading them to ingest large amounts of the drug [15]. This pattern of use implicates a potential risk of overdosing [16], [17]. Patients seeking medical attention after bath salts intoxications can display agitation, psychosis, tachycardia, and also hyperthermia, a commonly reported acute adverse effect of MDMA and beta-keto-amphetamine ingestion in humans [18]–[20].

The use of this substance in a chronic pattern may also be associated with more subtle long-term effects on brain neurotoxicity. However, there are discrepancies concerning its neurotoxicity in rodents. As with MDMA [21], it is possible that mephedrone could display a species-dependent neurotoxicity. Then, it is essential to compare results without losing sight of the species in which experiments are conducted. To date, authors found that mephedrone does not damage DA or 5-HT systems when administered to mice [22], [23]. Nevertheless, the studies did not extent the evaluation of DA to brain areas other than striatum or were performed with a drug exposure schedule not adjusted to mephedrone pharmacokinetics [24].

The aim of this paper was to investigate the neurotoxicity profile of mephedrone in mice, addressing some of the limitations in the literature. Most authors described the neurotoxic effects of methamphetamine three days after exposition [25] and those of MDMA seven days after [26]. We examined the neurotoxic injury induced by mephedrone at 3 and 7 days after finishing the exposition. Obtaining as much mechanistic information as possible regarding mephedrone, as well as on its neurotoxic effects, is of the essence. In this regard, we have evaluated the *in vivo* effect of this cathinone following different dosage schedule whilst complementing it by performing *in vitro* experiments. With regards to MDMA, it is described that the magnitude of the acute hyperthermic response plays a major role in determining the severity of the consequences of its misuse, in such a way that ingesting the drug in hot, crowded dance club conditions, increases the possibility of subsequent cerebral neurotoxic effects [27]. To simulate these usual conditions of drug exposure, the neurotoxicity studies with amphetamine-derivatives are usually performed at elevated ambient temperatures. Accordingly, present experiments were carried out at high room temperatures. This condition was not considered in previous published papers.

In the present study we used adolescent mice, a feature that correlates with young adult consumers. We demonstrate that mephedrone induces an injury at nerve endings in the frontal cortex at a schedule of drug exposure that mimics human “weekend consumption”.

## Experimental Procedures

### Drugs and reagents

Pure racemic mephedrone hydrochloride was synthetized and characterized by us as described previously [7]. The other drugs were obtained from Sigma-Aldrich (St. Louis, MO, USA). [^3^H]ketanserin, [^3^H]paroxetine, [^3^H]raclopride and [^3^H]WIN35428 were from Perkin Elmer Life Sci. (Boston, MA, USA). All buffer reagents were of analytical grade.

### Animals and ethics statement

The Experimental protocols were approved by the Animal Ethics Committee of the University of Barcelona, following the guidelines of the European Community Council (86/609/EEC). Male Swiss CD-1 mice (Charles River, Spain) aged 4–5 weeks (25–30 g) were used. Animals were housed at 22±1°C under a 12 h light/dark cycle with free access to food and drinking water.

### *In vivo* neurotoxicity assays

No information about the subcutaneous doses in humans is available. The only approach would be the doses used intranasally that can reach up to 125 mg in each insufflation. The typical amount of mephedrone consumed over an evening/night was about 0.5 to 1 gram, usually taken in doses of 100-200 mg every hour or two hours [28]. In our case a dose of 25 mg/kg in mice corresponds to a 2 mg/kg in an adult. This mice equivalent dose was calculated following the body surface area normalization method [29]. The interval of 2 h between doses was chosen according the mephedrone half-life [24].

Mice were administered subcutaneously (under the loose skin on their back) according the following schedules. Schedule 1: four doses of saline (5 ml/kg) or mephedrone (50 mg/kg) with a 2 h interval. Mephedrone doses higher than 4×50 mg/kg were not tested to avoid cardiotoxicity-associated complications [30]. Schedule 2: four doses of saline or mephedrone (25 mg/kg) with a 2 h interval. Schedule 3: three doses of saline or mephedrone (25 mg/kg) with a 2 h interval, for two consecutive days. Rectal temperatures were measured 45 min after the last dose by inserting into the rectum (1.5 cm) a lubricated, flexible rectal probe attached to a digital thermometer (Panlab, Barcelona, Spain). Rectal temperature was measured 40 s after insertion of the probe. During the expositions, the animals remained one per cage, were maintained in an ambient temperature of 26±2°C and were kept under these conditions until 1 h after the last daily dose. After initial results, we consider that schedule 3 was the most suitable and representative of weekend dosing. So, different parameters have been evaluated only in that schedule.

The doses of mephedrone used in this study were based on results from Angoa-Pérez et al. [22], who concluded that, in mice, a 4×20 mg/kg regimen does not elicit neurotoxicity in striatal DA nerve endings. Despite the importance of these results, it remains of the essence to assess other exposition regimens and their effects on several brain areas or neurotransmitters. In this sense, we focused on simulating weekend use patterns (prolonging the days of exposure but reducing the number of daily doses).

### Tissue sample preparation

Crude membrane preparation (collecting both synaptosomal and endosomal fraction) was prepared as described [31] with minor modifications. Mice were killed by cervical dislocation at 3 or 7 days after exposition. Hippocampus, striatum and frontal cortex were quickly dissected out and stored at −80°C until use. When required, tissue samples were thawed and homogenized at 4°C in 20 volumes of buffer (5 mM Tris-HCl, 320 mM sucrose) with protease inhibitors (aprotinin 4.5 µg/µl, 0.1 mM phenylmethylsulfonyl fluoride, and 1 mM sodium orthovanadate). The homogenates were centrifuged at 1,000 g for 15 min at 4°C. Aliquots of the resulting supernatants were stored at −80°C until use for Western blot experiments. The rest of the samples were resuspended and centrifuged at 15,000 g for 30 min at 4°C. The pellets were resuspended in buffer and incubated at 37°C for 5 min to remove endogenous neurotransmitters. The protein samples were recentrifuged. The final pellets were resuspended in the appropriate buffer and stored at −80°C until use in radioligand binding experiments. Protein content was determined using the Bio-Rad Protein Reagent.

We performed an additional experiment. Following decapitation, the brain of some animals was separated in the two hemispheres. In one of them we applied a cellular fractionation [32] to obtain a fraction with a high content in plasma membrane and a second one enriched with endosomes. In the other hemisphere we followed the standard method described above to obtain a crude membrane fraction.

### DA and 5-HT transporter density

The density of the DA transporter in striatal or frontal cortex membranes was measured by [^3^H]WIN35428 binding assays. These were performed in tubes containing 250 or 500 µl of 5 nM [^3^H]WIN35428 in phosphate-buffer and 50 or 100 µg of membranes, respectively. Incubation was done for 2 h at 4°C and non-specific binding was determined in the presence of 30 µM bupropion. All incubations were finished by rapid filtration under vacuum through Whatman GF/B glass fiber filters. Tubes and filters were washed rapidly three times with 4 ml of ice-cold buffer, and the radioactivity in the filters was measured by liquid scintillation spectrometry.

The density of the 5-HT transporter in the hippocampal and frontal cortex membranes was quantified by measuring the specific binding of 0.05 nM [^3^H]paroxetine after incubation with 150 µg of protein at 25°C for 2 h in a Tris-HCl buffer. Clomipramine (100 µM) was used to determine non-specific binding.

### Western blotting and immunodetection

A general western blotting and immunodetection protocol was used to determine tyrosine hydroxylase (TH) and tryptophan hydroxylase 2 (TPH2) levels. For each sample, 20 µg of protein was mixed with sample buffer (0.5 M TrisHCl, pH 6.8, 10% glycerol, 2% (w/v) SDS, 5% (v/v) 2-β-mercaptoethanol, 0.05% bromophenol blue), boiled for 5 min, and loaded onto a 10% acrylamide gel. Proteins were then transferred to polyvinylidene fluoride (PVDF) sheets (Immobilon-P; Millipore, USA). PVDF membranes were blocked overnight with 5% defatted milk in Tris-buffer plus 0.05% Tween-20 and incubated for 2 h at room temperature with a primary mouse monoclonal antibody against TH (Transduction Lab, Lexington, KY, USA) 1∶5000 or with a primary rabbit polyclonal antibody against TPH2 (Millipore, Billerica, MA, USA) 1∶1000 and anti-(phosphor-Ser-19)TPH2. After washing, membranes were incubated with a peroxidase-conjugated antimouse IgG antibody 1∶2500 or with a peroxidase-conjugated antirabbit IgG antibody (GE Healthcare, Buckinghamshire, UK) dil. 1∶5000. Immunoreactive protein was visualized using a chemoluminescence-based detection kit (Immobilon Western, Millipore, USA) and a BioRad ChemiDoc XRS gel documentation system (BioRad, Hercules, CA, USA). Scanned blots were analyzed using BioRad Image Lab software and dot densities were expressed as a percentage of those taken from the control. Immunodetection of beta-actin (mouse monoclonal antibody, 1∶2500) served as a control of load uniformity for each lane and was used to normalize differences due to protein content.

### 5-HT2A and D2 receptor density

The density of 5-HT2A receptors was measured in frontal cortex membranes one week after their exposure to schedule 3. Assays were performed in tubes containing 1 nM [^3^H]ketanserin and 100 µg of membranes. Incubation was carried out at 37°C for 30 min in a Tris–HCl buffer. Methysergide (10 µM) was used to determine non-specific binding. Assays to measure the density of D2 receptors in striatum membranes of the same animals were performed in tubes containing 2 nM [^3^H]raclopride and 50 µg of membranes. Incubation was carried out at 25°C for 1 h in a Tris–HCl buffer. Sulpiride (300 µM) was used to determine non-specific binding.

### Immunohistochemistry

Animals were anaesthetized with sodium pentobarbital (60 mg/kg, i.p.) and perfused through the heart with 4% paraformaldehyde in 0.1 M phosphate buffer (1 ml/g of body weight). Brains were removed and post-fixed for 2 h in the same solution, cryoprotected in 30% sucrose/phosphate buffer for 24 h and frozen in dry ice-cooled isopentane. Serial coronal sections (30 µm thick) through the whole brain were cut in a cryostat and collected in phosphate buffer. Free-floating sections were incubated for 15 min in H2O2 (0.3% phosphate buffer, 10% methanol). Thereafter, sections were incubated in a blocking solution (1% fetal bovine serum, 0.2 M glycine plus 0.5% Triton X100). After blocking with 10% normal serum and 0.2% bovine serum albumin, sections were rinsed and incubated overnight at 4°C using a monoclonal antibody against fibrillary acidic protein (GFAP, 1∶1000) (Dako, Denmark). Sections were washed and incubated with a biotinylated secondary antibody (1∶200 Sigma-Aldrich) for 2 h at room temperature. Afterwards sections were incubated with avidin-biotin-peroxidase complex (ABC; 1∶200; Vector, Burlingame, CA, USA). Peroxidase reaction was developed with 0.05% diaminobenzidine in 0.1 M phosphate buffer and 0.02% H2O2, and immunoreacted sections were mounted on gelatinized slides. Stained sections were examined under a light microscope (Olympus BX61).

### Neuronal cell cultures

Primary neuronal cultures of cerebral cortex were obtained from mouse embryos (E-16-18). The cerebral cortex was dissected, meninges were removed, and tissue was incubated for 20 min in trypsin (0.05%) at 37°C. Trypsin was inactivated with fetal bovine serum and tissue was triturated with a Pasteur pipette. Dissociated cells were washed with phosphate buffer containing 0.6% glucose and centrifuged at 500 g for 5 min. The cells were redissociated in Neurobasal medium (Invitrogen, Carlsbad, CA, USA) with 0.5 mM Lglutamine, sodium bicarbonate (0.04%) and 1 µg/ml penicillin and streptomycin, containing B27 supplement and 10% horse serum (HS). Neurons were plated at 0.4 million cells/ml in 96-well plates precoated with 1 mg/ml poly-L-lysine. Cultures were maintained at 37°C in an incubator with 5% CO2. 24 h after, cells were treated with arabinosylcytosine (10 µM) to prevent the growth of glial cells. The culture medium was changed after 4 days to Neurobasal medium with B27 without antioxidants. Before treating the cells, the HS concentration was reduced by half. The cultures were used for experiments after 8–9 days *in vitro* with different concentrations of mephedrone and different times of drug exposure.

### Cell viability

Cell viability was assessed using the MTT (3-(4,5-dimethylthiazole-2-yl)-2,5diphenyltetrazoliumbromide) method. This assay was carried out as described by Verdaguer et al. [33] with minor modifications. MTT was added to a final concentration of 250 µM and cells were incubated for 2 h [34]. Cell viability was expressed as a percentage of the absorbance measured in untreated cells.

### Forced swimming test (FST)

The immobility time in FST was measured by an observer blind to the exposition using the procedure described by Porsolt et al. [35]. Briefly, mice were placed individually in a glass cylinder (height 21 cm, diameter 12 cm) containing water at 25±1°C up to a height of 15 cm. Animals were randomly divided into two groups (12–16 animals/group) and treated with saline or mephedrone and tested 1, 3 or 7 days after schedule 3. Each animal was recorded for 6 min and the total period of immobility (in seconds) was measured. A mouse was judged to be immobile when it remained floating in water, making only the necessary movements to keep its head above the surface. Each mouse was used only once for each experimental session [36].

### Statistical analysis

All data are expressed as mean ± standard error of the mean (S.E.M.). Differences between groups were compared using one-way ANOVA or Student-t test for independent samples. Significant (p<0.05) differences were then analyzed by Tukey's post hoc test for multiple means comparisons where appropriate. Statistic calculations were performed using Graph Pad Instat (GraphPad Software, San Diego, USA). Analysis of concentration-viability curves was performed using non-linear regression (InvivoStat software [37]). Experimental values to calculate IC_50_ were in the linear region of the sigmoid curve (between 25 and 85% of the maximum effect).

## Results

### Lethality

Initial experiments were carried out with 6 animals per cage. With this condition, lethality was of about 70%. Thereafter, all experiments carried out in this study were performed with a single animal per cage. The number of fatalities of mephedrone-treated mice was similar in schedule 2 (8.33%) and 3 (10.17%), and slightly higher in schedule 1 (14.28%). It is important to note that all mephedrone schedules induced the occurrence, in some animals, of a stereotypy (repeated self-licking of the ventral base of the neck) that continued with self-bites and led to the appearance of injuries in that zone.

### Effect of mephedrone on body temperature and weight gain

In the present study, drug expositions were performed in animals housed singly at a high room temperature. All mephedrone schedules induced a significant increase in body temperature. Hyperthermia was apparent in schedule 1 (saline: 36.4±0.1°C; mephedrone: 38.1±0.1 p<0.001) and schedule 2 (saline: 36.7±0.1°C; mephedrone: 37.4±0.2°C p<0.01). Results obtained from schedule 3 showed that mephedrone–induced hyperthermia was more intense after the last dose in the first day of exposition (saline: 36.7±0.1°C; mephedrone 38.1±0.1, first day; vs. saline: 36.4±0.1°C; mephedrone 37.5 ±0.3, second day, p<0.001). Mephedrone exposure slowed the weight gain compared with saline-treated animals (Table 1). Moreover, 3 days after finishing all schedules, the animals recovered their body weight (data not shown).

**Table 1:** Effect of mephedrone on weight gain.

	Schedule		
Group	1	2	3
Saline	0.71±0.14	0.84±0.22	0.77±0.21
Mephedrone	−0.99±0.14^a^	−0.98±0.16^a^	−1.23±0.20^b^

Data are expressed (mean ±S.E.M) as the difference in body weight, in grams, between the end (24 h after the last dose) and the beginning (prior to the first dose) of the exposure.

a sig. diff. (*p*<0.01) vs. Saline.

b sig. diff. (*p*<0.001) vs. Saline.

### Effect of mephedrone on different *in vivo* markers of DA and 5-HT neurotoxicity

**Figure 1:**
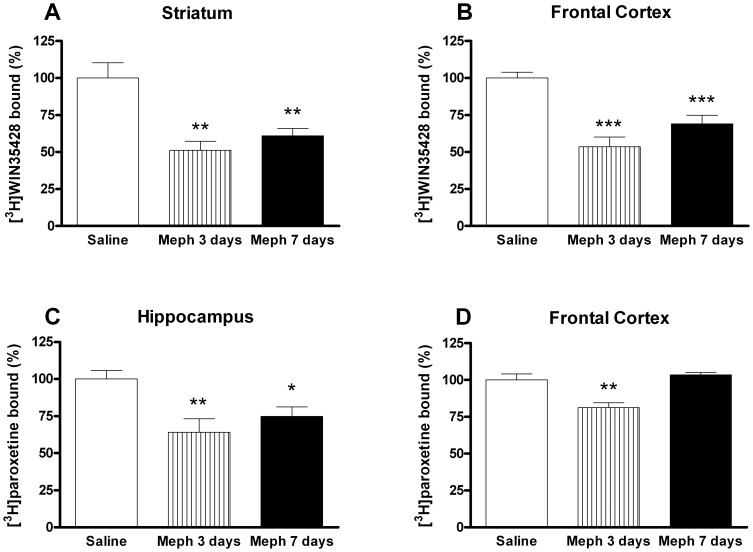
Effect of a mephedrone exposure (4 doses of 50 mg/kg, s.c. at 2 h interval) on dopamine transporter density, measured as [^3^H]WIN35428 binding in mouse striatum (A), or frontal cortex (B) and 5-HT transporter density, measured as [^3^H]paroxetine binding, in hippocampus (C) and frontal cortex (D). Results are expressed as mean±S.E.M. from 8–10 animals. *p<0.05; **p<0.01 and ***p<0.001 vs. saline.

Schedule 1: At 3 and 7 days post-exposition, mephedrone induced a significant loss in DA reuptake sites of about 50% in mouse striatum and frontal cortex membranes (Fig. 1A and 1B). Additionally, mephedrone-exposed mice showed a transient decrease in [^3^H]paroxetine binding in the frontal cortex that disappeared four days later (Fig. 1D). By contrast, the decrease of [^3^H]paroxetine binding in the hippocampus was apparent both 3 and 7 days after exposition (Fig. 1C).

**Figure 2:**
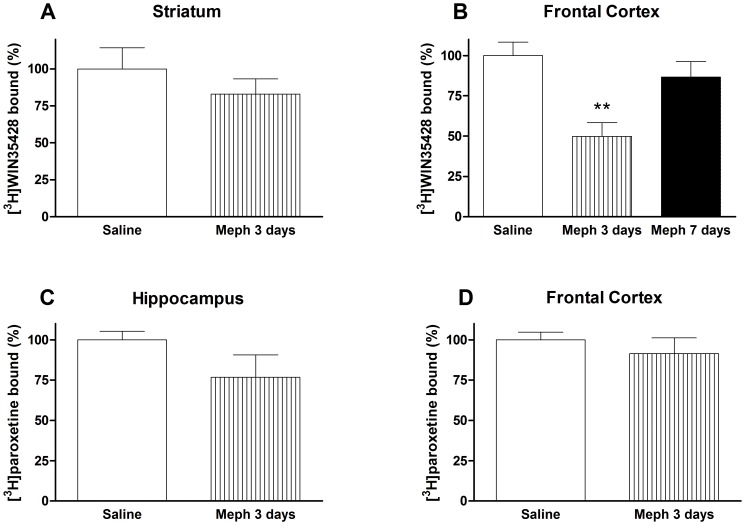
Effect of a mephedrone exposure (4 doses of 25 mg/kg, s.c. at 2 h interval) on dopamine transporter density, measured as [^3^H]WIN35428 binding in mouse striatum (A), or frontal cortex (B) and 5-HT transporter density, measured as [^3^H]paroxetine binding, in hippocampus (C) and frontal cortex (D). Results are expressed as mean±S.E.M. from 8–10 animals. **p<0.01 vs. saline.

Schedule 2: 3 days after the drug regimen, the frontal cortex was the only area where a marker showed a significant decrease. For this reason, the specific binding of [^3^H]WIN35428 in this area, was also evaluated 4 days later. At this time, DA transporter density returned to basal levels (Fig. 2B). Mephedrone did not modify [^3^H]WIN35428 binding in the striatum (Fig. 2A) or the [^3^H]paroxetine binding neither in the hippocampus nor in the frontal cortex (Fig. 2C and 2D).

**Figure 3:**
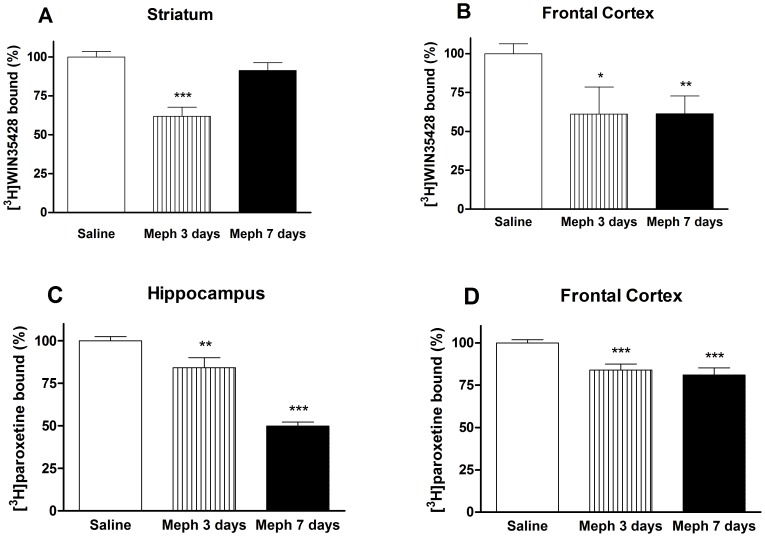
Effect of a mephedrone exposure (3 doses of 25 mg/kg, s.c. at 2 h interval for 2 days) on dopamine transporter density in mouse striatum (A), or frontal cortex (B) and on 5-HT transporter density in hippocampus (C) and frontal cortex (D). Results are expressed as mean±S.E.M. from 8–10 animals. *p<0.05; **p<0.01 and ***p<0.001 vs. saline.

Schedule 3: The suppressive effect of mephedrone on striatal [^3^H]WIN35428 binding was transient and returned later to basal values (Fig. 3A). However, in frontal cortex, the same marker suffered a significant loss (about 40%) evident 3 and 7 days post-exposition (Fig. 3B). Because these results pointed to a real injury more than to a transient regulation, we investigated the expression of the TH. We found a relationship between the decrease in the [^3^H]WIN35428 specific binding and the decrease in enzyme expression in the frontal cortex (saline: 100.00±2.46%; mephedrone: 60.04±9.34% p<0.01, 3 days after exposition and 55.30±9.11% p<0.001, 7 days after exposition).

To characterize the recovery of the DA transporter marker in striatum, we assessed radioligand binding in the plasma membrane and in the endosomal fractions. Mephedrone elicited a reduction in [^3^H]WIN35428 binding in the plasma membrane (saline: 100.00±6.49%; mephedrone: 65.80±9.00% p<0.01) and the corresponding increase in the endosome fraction (saline: 100.00±10.51%; mephedrone: 127.43±5.94% p<0.05). Consequently, when a redistribution of transporter occurred, we did not find a loss of radioligand binding in the crude membrane preparation.

**Figure 4:**
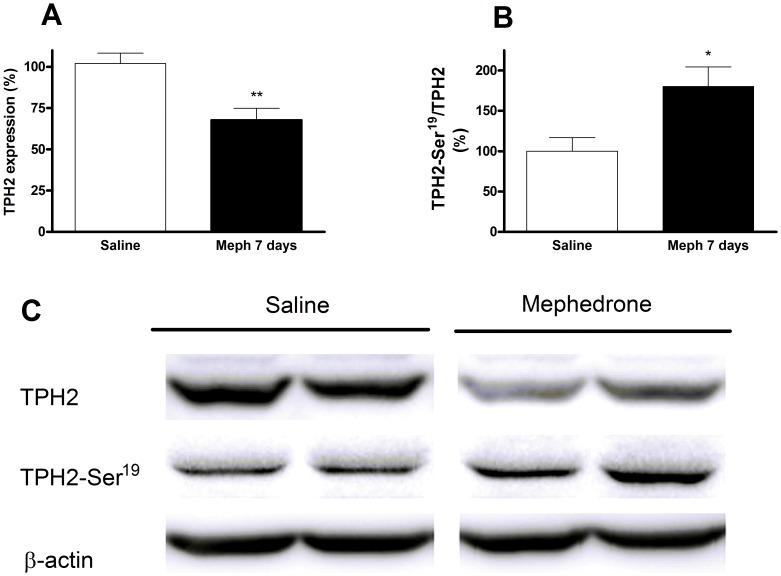
Effect of a mephedrone exposure (3 doses of 25 mg/kg s.c. at 2 h interval for 2 days) on total tryptophan hydroxylase TPH2 expression (A) and its phosphorylated form (B) in hippocampus. Panel C shows a representative Western blot. Results are expressed as mean±S.E.M. from 8–10 animals. *p<0.05 and **p<0.01 vs. saline.

**Figure 5:**
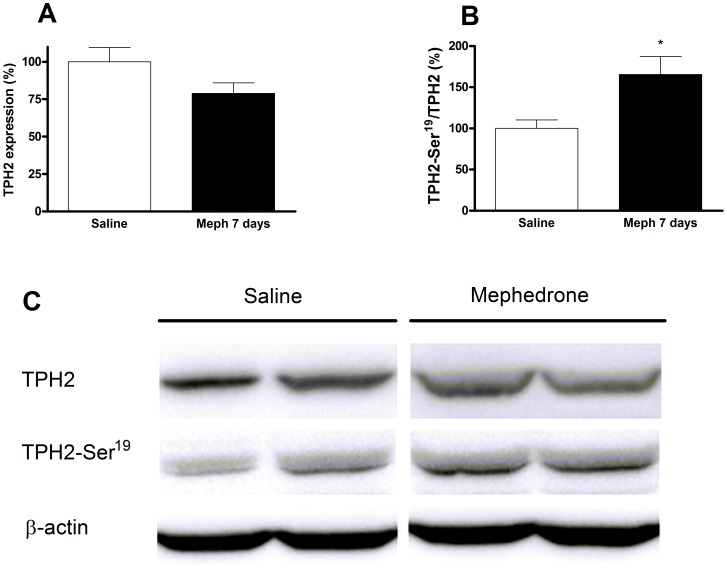
Effect of a mephedrone exposure (3 doses of 25 mg/kg s.c. at 2 h interval for 2 days) on total tryptophan hydroxylase 2 expression (A) and its phosphorylated form (B) in frontal cortex. Panel C shows a representative Western blot. Results are expressed as mean±S.E.M. from 8–10 animals. *p<0.05 vs. saline.

The reduction of 5-HT terminal markers in mephedrone-treated animals was sustained and significant in the frontal cortex (Fig. 3D), and especially pronounced (50%) in the hippocampus after 7 days (Fig. 3C). Consequently, we analyzed another biochemical marker of terminal integrity, TPH2, and its Ser-19 phosphorylated form. In hippocampus, the decrease of [^3^H]paroxetine binding runs in parallel with a decrease of the total TPH2 (Fig. 4A). In frontal cortex, this expression was lower but not significant (Fig. 5A). In both experiments, the shape of the TPH2 band obtained from mephedrone-treated animals shows differences that can be attributed to a protein modification. As protein phosphorylation in Ser-19 has been described as a frequent mechanism that regulates TPH2 function, we proceeded to determine it. In both brain areas, the remaining enzyme was phosphorylated in the mephedrone group with respect to saline (Fig. 4B, 4C, 5B, 5C).

### Effect of mephedrone on astroglial activation

**Figure 6:**
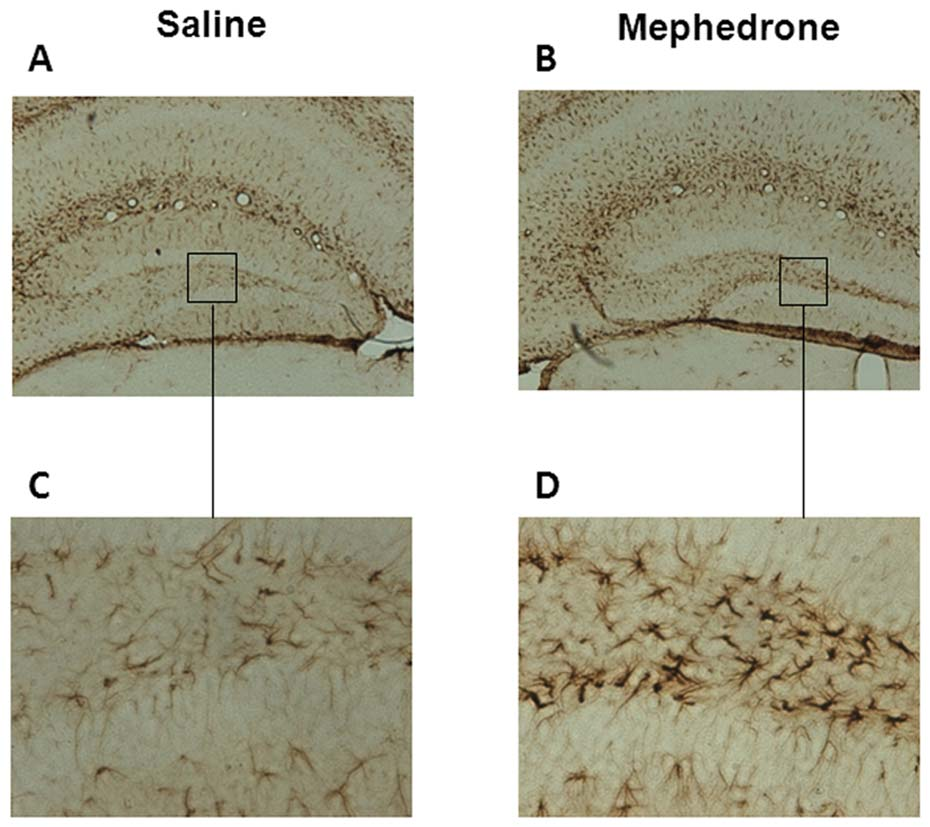
Representative hippocampal expression of glial fibrilliary acidic protein (GFAP). Sections of the dentate gyrus (×4 A, B; ×20, C, D) from mice exposed to saline (A, C) or mephedrone (3 doses of 25 mg/kg given subcutaneously for 2 days) (B, D). The animals were sacrificed 7 days after the last dose.

To assess the presence of astroglial activation, immunohistochemistry studies were carried out with the glial-specific marker, GFAP. There were no signs of striatal or cortical astroglial activation in mephedrone-treated animals. In the hippocampus, some astrocytes with the typical stellate morphology were observed in control animals, but an apparent increase in GFAP immunoreactivity could be seen in the dentate gyrus of the hippocampus in the mephedrone group, implying reactive astrocytes (Fig. 6).

### Depressant-like effect of mephedrone

**Figure 7:**
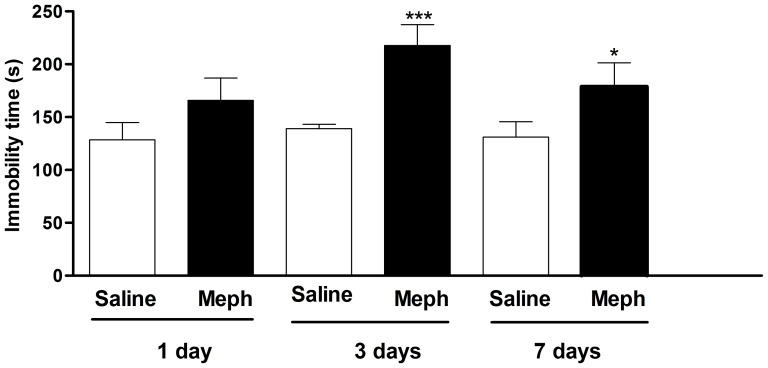
Effect of mephedrone on immobility time in mouse forced swim test. Animals were randomly divided into two groups (12–16 animals/group) and administered subcutaneously with saline (5 ml/kg) or mephedrone (3 doses of 25 mg/kg for 2 days) and tested 1, 3 or 7 days after exposure. Each animal was recorded for 6 min and the total period of immobility (in seconds) was registered. Each mouse was used only once for each experimental session. Each bar represents mean±S.E.M. immobility time. *p<0.05 and ***p<0.001 vs saline (one-way ANOVA and post hoc Tukey test).

In schedule 3, mephedrone significantly increased the immobility time in the FST as compared with saline group (F3,44 = 5.509, p<0.01). Post hoc Tukey's means comparison test demonstrated that 3 days post-exposition mephedrone significantly increased the immobility time (saline: 125.43±11.83 s; mephedrone: 217.83±19.55 s p<0.001). This increase was still significant 7 days post-exposition (178.80±22.45 s p<0.05). Moreover, mephedrone exposure failed to influence the immobility time in FST 24 h after the last administration (Fig. 7).

### Effect of mephedrone on D2 and 5-HT2A receptor density

To determine the involvement of striatal DA D2 receptors, we measured [^3^H]raclopride binding in this brain area. Mephedrone (schedule 3) decreased the number of these receptors in mouse striatum (saline: 100.00±6.49%; mephedrone: 79.86±5.46% p<0.05, 3 days after exposition and 78.57±7.42% p<0.05, 7 days after exposition). Similarly, mephedrone-exposed animals showed a decrease in the number of 5-HT2A receptors in frontal cortex (saline: 100.00±6.28%; mephedrone: 67.41±3.07% p<0.001) and hippocampus (saline: 100.00±8.45%; mephedrone: 71.70±4.02% p<0.01), 3 days after exposition. However, the density of these receptors returned to basal values on day 7 (98.81±4.01% and 98.93±4.10%, respectively).

### Effect of mephedrone on cultured cortical neuron viability

The exposition of mouse cortical cultured cells to various concentrations of mephedrone (from 80 µM to 1 mM) for 24 h or 48 h caused a concentration-dependent decrease in metabolically active cells, as assessed by the MTT assay. The calculated LD50 value for mephedrone after 24 h of incubation was 242.72±40.66 µM which was higher (p<0.01) to that obtained after 48 h of drug exposure (115.94±16.58 µM).

## Discussion

The easy availability of cathinones and their initial status as legal highs may have contributed to their increasing popularity as drugs of abuse. Because of the relatively short history of the use of cathinones as recreational drugs, their effects among long-term users have yet to be determined. Based on its structural similarity to well established neurotoxic psychostimulants such as methamphetamine and MDMA, it was hypothesized that mephedrone would exert neuronal damage. However, there are important discrepancies concerning the neurotoxicity induced by cathinones [8], [22], [38]. The inconsistent results could be attributed to differences in species, dosage, administration route or ambient temperature.

Some cases of aggressive behavior, even cannibalism, as a consequence of exposure to new designer drugs have been recently reported in the media. However, these cases have been poorly documented [39]. In the present study, all mephedrone schedules induced the appearance of initial stereotypy consisting in repeated self-licking that was followed by aggressive behavior which leads to self-injuries. This is an especially important factor to be taken into account, seeing as it required animals to be housed individually. It is a feature described for the first time, but it has been also reported following high doses of methamphetamine or d-amphetamine [40]–[41], but not MDMA involving DA and 5-HT system in this abnormal behavior. Fantegrossi et al. [42] reported self-injurious behavior following methylenedioxypryrovalerone administration only at high, but not normal, ambient temperature, suggesting a potential role of the ambient temperature in this behavior. Further studies are needed to determine the mechanism implicated in this feature induced by mephedrone and its relationship with the exposed dose.

Temporal development of body weight of mice was studied to ascertain an easily measurable effect of mephedrone. Like other amphetamines, animals treated with mephedrone experienced a reduction in weight gain that was only transient seeing as values returned to basal values 3 days after finishing the corresponding exposure.

Hyperthermia is a commonly reported acute adverse effect of amphetamines [43] and beta-keto-amphetamine ingestion in humans [19]. Rodent exposure to cathinones also cause significant increases in core temperature [8], [23], [44], [45]. The temperature has been examined during administration of multiple doses of the drug. Probably the strain of mouse, the dose and the ambient temperature can influence the size and direction of the hyperthermic response observed in the present experiments. Although lethal hyperthermia was not observed at the assessed dosages in mice, present results demonstrate that at high ambient temperatures, mephedrone impaired the thermoregulatory response; this effect persisted throughout the two day exposition. Nevertheless further research is required in order to characterize whether the role of hyperthermia is complementary or essential in the advent of mephedrone-induced neurotoxicity.

Several studies established a controversy concerning the neurotoxic effect of mephedrone in DA and 5-HT systems. Angoa-Pérez and co-workers [22] concluded that mephedrone administration to C57/BL6 mice at doses up to 40 mg/kg did not damage DA nerve endings in the striatum. In that study, no information regarding the ambient temperature is provided. Moreover, Hollander et al. [23] demonstrated that mephedrone exposure (30 mg/kg, twice daily for 4 days) to rats and mice resulted in no significant changes in brain monoamine levels. Conversely, Hadlock et al. [38] reported a rapid decrease in DA and 5-HT transporter function after mephedrone administration (four doses of 10 or 25 mg/kg) to Sprague-Dawley rats at an ambient temperature of 27°C. However, other investigators have failed to find any persistent neurochemical impact of mephedrone dosing with long duration dosing protocols [46].

In the effort to model recreational mephedrone use, we considered it appropriate to simulate the widespread practices of “stacking” (taking multiple doses at once in order to increase the desired effect and/or overcome tolerance from prior use) and “boosting” (taking supplemental doses over time in order to maintain the drug effect). For this reason, we chose to administer multiple doses of mephedrone during each exposition day. In schedule 3 we repeated the exposition the next day, simulating a pattern of a recreational weekend use.

It is important to note that we corroborated that the crude membrane preparation used in the present experiments collects both the synaptosomal membrane and the endosomal fraction; consequently it is not possible to ascertain whether or not a transporter redistribution was taking place when observing no decrease in radioligand binding in crude membrane preparations. We demonstrate that mephedrone, administered at doses (i.e. schedule 1) that mimic a high exposure in humans (around 1.5 g/session) [47], induced an important decrease in DA transporter density in mouse striatum and frontal cortex membranes that persisted 7 days after exposition. This effect was accompanied by a significant loss of 5-HT transporters in the hippocampus. However, at these high doses, acute cardiovascular toxicity of mephedrone is likely [30] and probably outweighs neurotoxic effects.

The subsequent experiments were carried out at a lower dose (25 mg/kg). Schedule 2 dosage schedule only elicited a transient decrease in cortical DA transporter, suggesting a temporary regulatory effect. Moreover, no significant loss of 5-HT transporter in the frontal cortex or the hippocampus was found.

Schedule 3 (three doses of 25 mg/kg for two consecutive days) can be considered the most representative because it is closest to the typical weekend consumption pattern. After this exposure, mephedrone induced loss of DA and 5-HT transporters that were especially apparent in the frontal cortex and the hippocampus respectively. The monoamine deficit induced by this schedule was also characterized by a significant decrease of each enzymatic marker that correlated with the decrease in radioligand binding. TH and TPH catalyze the first and rate-limiting step in the biosynthesis of DA and 5-HT respectively. The isoform TPH2 is responsible for 5-HT biosynthesis in the brain. Post-translational modifications have been shown to regulate the protein function. The enzyme is known to be phosphorylated on Ser-19 by both protein kinase A and Ca2+/calmodulin dependent protein kinase II *in vitro*. This modification results in increased stability and activity [48]. The decrease in transporter binding and enzyme levels, jointly with astrogliosis, point to an injury at the nerve endings; the increase in Ser-19TPH2 in mephedrone-treated animals seems to reflect a compensatory mechanism in the undamaged 5-HT terminals

The recovery of DA transporter levels in mouse striatum after 7 days of exposition raises the question on whether the decrease in [^3^H]WIN35428 binding observed 3 days after exposition is an effect of biochemical down-regulation in the absence of tissue damage rather than being reflective of an injury. The non-significant increase in astroglial activation observed in this area was consistent with the absence of terminal injury and suggests that the DA transporter gene expression may be negatively regulated by mephedrone exposure. This is consistent with previous results reporting that MDMA acts on 5-HT transporter gene expressions [49]. Moreover, although DA transporter density returns to basal values in crude membrane preparations 7 days after exposition, results point to changes in DA levels in the striatal synapses, seeing as there is a significant redistribution of this transporter. A significant increase in transporter density is observed in the endosomal fraction, together with a reduction in membrane expression. Astrocytes stabilize and maintain homeostatic repair of tissues and contribute to early wound repair [50]. In the present study, mephedrone-exposed animals showed an increase in GFAP immunoreactivity in the dentate gyrus of the hippocampus, which confirms the injury in this area.

Low 5-HT levels in the synapse have been linked to various psychiatric disorders, including depression. In our study, mephedrone increased the immobility time in the forced swim test, which indicates an increase in stress-related depressive behavior. This effect was evident 3 and 7 days after exposition, and correlates with the reduction of the neurochemical parameters. This is in accordance with results from McGregor et al. [51] who demonstrated that MDMA-treated animals show higher immobility and fewer active escape attempts in the forced swimming model. To our knowledge, present results provide the first preclinical data on this matter, and suggest that mice exposed to a stacking and boosting regime of mephedrone could be more prone to depressive-like symptoms.

Recently, it has been established that methamphetamine exposure dysregulates D2mediated DA transmission in the striatum [52]; Vidal-Infer et al. [53] also demonstrated that, in adolescent mice, striatal DA D2 receptors are involved in the rewarding properties of MDMA. Accordingly, we have studied the alteration in the striatal density of these receptors after the third mephedrone schedule. The density of D2 receptors remained below control values both 3 and 7 days after mephedrone exposition, pointing to an increase in the susceptibility of these animals to drug addiction [54]. Moreover, we found a significant transient decrease in the number of 5-HT2A receptors 3 days after exposition, which can be reflective of a neuroadaptative response to the increase in 5-HT release induced by mephedrone. These results are in agreement with those reported by Scheffel et al. [55] who demonstrated that the repeated administration of MDMA causes transient down-regulation of 5-HT2 receptors, which are predominantly abundant in the frontal cortex.

We performed *in vitro* studies in cortical cultured cells in order to assess a theoretical concentration that could be injurious on neuron viability, which is known to be quite high for most amphetamines. In these cells we found a concentration-dependent cytotoxic effect of mephedrone in neuronal viability, which increases significantly with respect to time of drug exposure. It must be pointed that this cytotoxicity is higher than that of MDMA in primary cultures of hippocampal neurons [56]. According to our previous pharmacokinetic data of mephedrone in rats [24], an i.v. dose of 10 mg/kg leads to an extrapolated mephedrone blood concentration of about 5.6 µM. Then, a dose of 25 mg/kg would correspond to a mephedrone concentration of about 14 µM that is about 15 times lower than the LD_50_ in cultured cortical cells. It is important to note that in the present study, when exposing mice to 25 mg/kg of mephedrone no neuronal death is suspected, as there were no signs of striatal or cortical astroglial activation in mephedrone-exposed animals. Only, some reactive astrocytes were appreciated in the dentate gyrus. Additionally, we did not observe microglial activation (data not shown). Results from cortical cultured cells seem to point in the same direction because neuronal death is obtained at higher concentrations than those reached *after in vivo* exposure. Nonetheless, as occurs with MDMA, we cannot exclude the possibility that some of its metabolites may cause toxic effects of this nature, since the *in vivo* metabolic pathway of mephedrone is similar to that of MDMA [57].

Finally, our results demonstrate that mephedrone-induced brain injury differs according to exposition schedule (dose, number of administrations and intervals). Neuronal toxicity elicited by mephedrone is apparent when administered in 2 h intervals. This is in accordance with our previous paper characterizing the pharmacokinetics of mephedrone [24]. Using this dose interval and repeated doses for 2 days, in an elevated ambient temperature, we found a loss of frontal cortex dopaminergic and hippocampal serotoninergic neuronal markers together with astrogliosis, pointing to the presence of injuries at nerve endings. A compensatory mechanism runs in parallel with this process, including an increase in the phosphorylated form of TPH2, a different subcellular distribution of DA transporter, and a decrease in D2 receptors.
